# Comparison of two intraductal brush cytology devices for suspected malignant biliary strictures: randomized controlled trial

**DOI:** 10.1007/s00464-023-09916-9

**Published:** 2023-02-27

**Authors:** Myrte Gorris, Nadine C. M. van Huijgevoort, Paul Fockens, Sybren L. Meijer, Joanne Verheij, Rogier P. Voermans, Roy L. J. van Wanrooij, Selma J. Lekkerkerker, Jeanin E. van Hooft

**Affiliations:** 1 Gastroenterology and Hepatology, Amsterdam UMC Location University of Amsterdam, Amsterdam, The Netherlands; 2 Amsterdam Gastroenterology Endocrinology Metabolism, Amsterdam, The Netherlands; 3 Cancer Center Amsterdam, Amsterdam, The Netherlands; 4 Gastroenterology and Hepatology, Amsterdam UMC Location Vrije Universiteit Amsterdam, Amsterdam, The Netherlands; 5 Department of Pathology, Amsterdam UMC Location University of Amsterdam, Amsterdam, The Netherlands; 6 Department of Gastroenterology and Hepatology, Leiden University Medical Center, Leiden, The Netherlands

**Keywords:** Biliary tract neoplasms, Endoscopic retrograde cholangiopancreatography, Clinical pathology, Pancreatic neoplasms, Sensitivity, Specificity

## Abstract

**Background:**

Endoscopic retrograde cholangiopancreatography (ERCP) with biliary brush cytology is commonly used to diagnose malignant pancreatobiliary strictures. This trial compared the sensitivity of two intraductal brush cytology devices.

**Methods:**

A randomized controlled trial in which consecutive patients with suspected malignant, extrahepatic biliary strictures were randomized (1:1) to a dense or conventional brush cytology device. Primary endpoint was sensitivity. Interim analysis was conducted after 50% of the patients completed follow-up. Results were interpreted by a data safety monitoring board.

**Results:**

Between June 2016 and June 2021, 64 patients were randomized to the dense (27 patients, 42%) or conventional brush (37 patients, 58%). Malignancy was diagnosed in 60 patients (94%) and benign disease in 4 patients (6%). Diagnoses were confirmed by histopathology in 34 patients (53%), cytopathology in 24 patients (38%), and clinical or radiological follow up in 6 patients (9%). Sensitivity of the dense brush was 50%, compared to 44% for the conventional brush (*p* = 0·785).

**Discussion:**

The results of this randomized controlled trial showed that the sensitivity of a dense brush is not superior to a conventional brush for diagnosing malignant extrahepatic pancreatobiliary strictures. This trial was prematurely ended for reasons of futility.

**Trial registration:**

Netherlands Trial Register number; NTR5458.

**Supplementary Information:**

The online version contains supplementary material available at 10.1007/s00464-023-09916-9.

Malignant pancreatobiliary strictures are most commonly caused by pancreatic ductal adenocarcinoma (PDAC) and distal cholangiocarcinoma (CCA). Due to the late presentation in advanced stages of disease, only 20% of the patients can be treated with curative intent. Chemotherapy is the mainstay of treatment as it prolongs overall survival in both the curative and the palliative setting [1]. In addition, neoadjuvant therapy is increasingly administered prior to surgical resection since it increases overall survival [2–4]. Prior to initiation of (chemo) therapy, it is imperative to obtain pathological proof of malignancy. Diagnostic tools with high sensitivity are therefore crucial since, considering the aggressive course of disease, inconclusive samples may lead to delay or even annulment of (chemo) therapy. Endoscopic retrograde cholangiopancreatography (ERCP) is performed in the majority of patients with malignant pancreatobiliary strictures to ensure adequate biliary drainage and can easily be combined with biliary brush cytology to obtain a tissue diagnosis. Several systematic reviews reported that conventional biliary brush cytology has a specificity of nearly 100%. Unfortunately, sensitivity remains poor and ranges from 42–45% [5, 6]. Few studies suggested that the sensitivity of a dense biliary brush cytology device (the Infinity® brush), which is designed to maximize tissue acquisition by using a combination of stiff and soft bristels, is higher (60–78%) when compared to conventional cytology brush devices [7, 8]. These results are promising and have significant clinical value, since higher sensitivity will result in minimization of false-negative test results and thereby minimize treatment delay. Nevertheless, a possible disadvantage of the dense brush cytology device is its larger diameter and, as a consequence, the necessity to perform concomitant sphincterotomy. Both the sphincterotomy and the diameter of the device might lead to a higher rate of post-procedural adverse events (i.e., pancreatitis, bleeding, perforation, cholangitis, or cholecystitis). The aim of this randomized controlled trial was therefore to compare the sensitivity between a dense versus a conventional brush cytology device in patients with suspected malignant, extrahepatic pancreatobiliary strictures.

## Materials and methods

### Study design

We performed a single-blinded, randomized controlled trial to prove superior sensitivity of the dense Infinity® brush (US Endoscopy, Northeast Ohio, USA. CE 02,112) over the conventional RX cytology® brush (Boston scientific Corporation, Marlborough, MA, USA. CE 616,288) for diagnosing malignant, extrahepatic biliary strictures. The study was performed in the Amsterdam University Medical Centers (location Academic Medical Center and VU Medical Center, Amsterdam), a tertiary care centre in the Netherlands. The study was approved on January 14th, 2016 by the local institutional review board of the Academic Medical Center (approval number METC 2015_240). The independent data safety monitoring board (DSMB) consisted of two gastroenterologists and a clinical epidemiologist. The DSMB evaluated the results of the interim analysis (after 50% of the patients completed follow-up, *n* = 56) for adverse events and futility. This trial was registered in the Netherlands Trial Register (NTR5458) and can be consulted via https://www.trialregister.nl/trial/5234.

### Participants

All consecutive patients ≥ 18 years with a suspected malignant, extrahepatic biliary stricture who underwent ERCP and had an indication to obtain a cytological sample via biliary brush cytology were eligible for inclusion. Patients provided written informed consent prior to the procedure. Exclusion criteria were: Intrahepatic or hilar biliary obstruction (defined as biliary stricture located within 2 cm of the hilum), failed biliary cannulation, contraindication for sphincterotomy, and the absence of a malignant stricture during ERCP. During the course of this study, another randomized trial on the value of endoscopic sphincterotomy prior to fully covered self-expandable metal stent placement for the prevention of pancreatitis was conducted [9]. If patients were randomized to the ‘no sphincterotomy’ group in this trial, they were excluded from the current trial based on a contraindication for sphincterotomy.

### Randomization and masking

After biliary cannulation was obtained, patients were randomly assigned (1:1) by the coordinating investigator to either the intervention group (dense brush) or the control group (conventional brush) with the use of sealed opaque envelopes. The randomization sequence was computer-generated before trial commencement by SL. Patients were enrolled and assigned to trial groups by the coordinating investigators (SL, NvH, MG). Participants were masked to group assignment (they were not told which group they were allocated to), whereas the endoscopist was not masked to the outcome of randomization. Pathologists were blinded to group assignment. The study coordinator was not blinded to treatment allocation during the assessment of the outcomes and the analyses of the study data. The members of the DSMB were blinded to group allocation.

### Procedures

ERCP procedures were performed by or under direct supervision of a dedicated interventional endoscopist using standard techniques. Because of the diameter (9 French) of the dense brush, all patients in the intervention group underwent concomitant sphincterotomy. In the control group, sphincterotomy was performed at the discretion of the endoscopist. Cytology was obtained by pulling the brush back and forth through the stricture for 10 times in both groups. Hereafter, the brush was covered with the sheet and pulled back. Brushes were placed in a standard cytology vial and the cover of the brush was flushed in the vial to optimize cellular yield. The samples were evaluated by pancreatobiliary-dedicated pathologists as part of standard care. Deoxyribonucleic acid (DNA) mutation analysis, immunohistochemical staining, and central reading of the brush cytology samples were not standardly performed. Brush cytology samples were classified according to the Bethesda score [10]. Follow-up was performed 5–7 days, 28–30 days and 6 months after the procedure by telephone or based on clinical data from the electronic patient file.

### Outcomes

The primary outcome was sensitivity, defined as brush cytology specimen showing at least suspicion of malignancy (Bethesda ≥ 4) in patients with malignant diagnosis, as confirmed by histopathology results (surgical specimen or biopsy of either the primary mass or distant metastasis) or cytopathology results (ultrasound-or computed tomography (CT)-guided fine needle aspiration of distant metastasis or endoscopic ultrasonography-guided fine-needle aspiration (EUS-FNA) of the primary mass) showing at least suspicion of malignancy, or clinical and/or radiological follow-up. Secondary endpoints were specificity (defined as Bethesda ≤ 3 in patients with benign disease), positive predictive value (PPV, defined as the rate of true-positive results among all positive tests), negative predictive value (NPV, defined as the rate of true-negative results among all negative test results), and adverse events. Adverse events were classified according to the Cotton criteria (pancreatitis, gastro-intestinal bleeding, perforation, and cholangitis) [11, 12]. Cholecystitis was classified according to the 2018 Tokyo guidelines [13]. Adverse event severity was classified according to the Clavien-Dindo classification [14]. Clinically relevant gastro-intestinal bleeding, pancreatitis, cholangitis, cholecystitis, and stent dysfunction which required hospitalization were considered procedure-related adverse events.

### Statistical analysis

Sample size calculation was based on the results of a meta-analysis which evaluated the sensitivity of conventional brush cytology in 1556 patients from 16 studies [6]. In total, 55% of patients had malignant disease and sensitivity was 42%. Diagnostic performance of the more dense brush was evaluated in 2 studies that reported a sensitivity of 75–78% [7, 15]. Therefore, we assumed a sensitivity of 42% in the conventional brush group and a 30% increase in sensitivity in the dense brush group. With the use of a Chi-square test, with an 0.05 two-sided significance level and 80% power to detect a 30% difference, sample size was estimated at 42 patients in each group. Since we also included patients with previous pathologically proven malignancy and the results of the previously mentioned meta-analysis showed that 55% of patients with suspected malignant biliary obstruction have final malignant disease, we expected the prevalence of malignancy in our cohort to be 75%. Thus, sample size was set on 112 patients in total (56 patients per group). Continuous variables were expressed as medians with its corresponding interquartile range (IQR). Categorical data were presented with proportions and percentages. Differences between groups were calculated by using the Mann–Whitney U test for non-parametric continuous data. The chi-square test (or Fisher’s exact test if appropriate) was used to compare categorical variables. Data were analyzed with the use of IBM SPSS Statistics version 26 (IBM Corp. Released 2019. IBM SPSS Statistics for Windows, Version 26.0. Armonk, NY: IBM Corp). A *p*-value of < 0.05 was considered statistically significant.

#### Early termination

According to protocol, the trial would be terminated after the interim analysis in case of a statistically significant higher rate of adverse events in the intervention group without a clinically relevant increase in sensitivity. Furthermore, rules for efficacy (according to the Haybittle-Peto rule) and futility (based on the O’Brien & Fleming method) were incorporated in the study protocol. The results of the interim analysis at 50% (*n* = 56) accrual were evaluated by the DSMB. Inclusion of participants was continued until the DSMB statement was revealed. Since the results of the interim analysis met the definition of futility and a small difference in sensitivity was judged by the DSMB as having little clinical relevance, the DSMB recommended that the trial should be terminated.

## Results

**Fig. 1:**
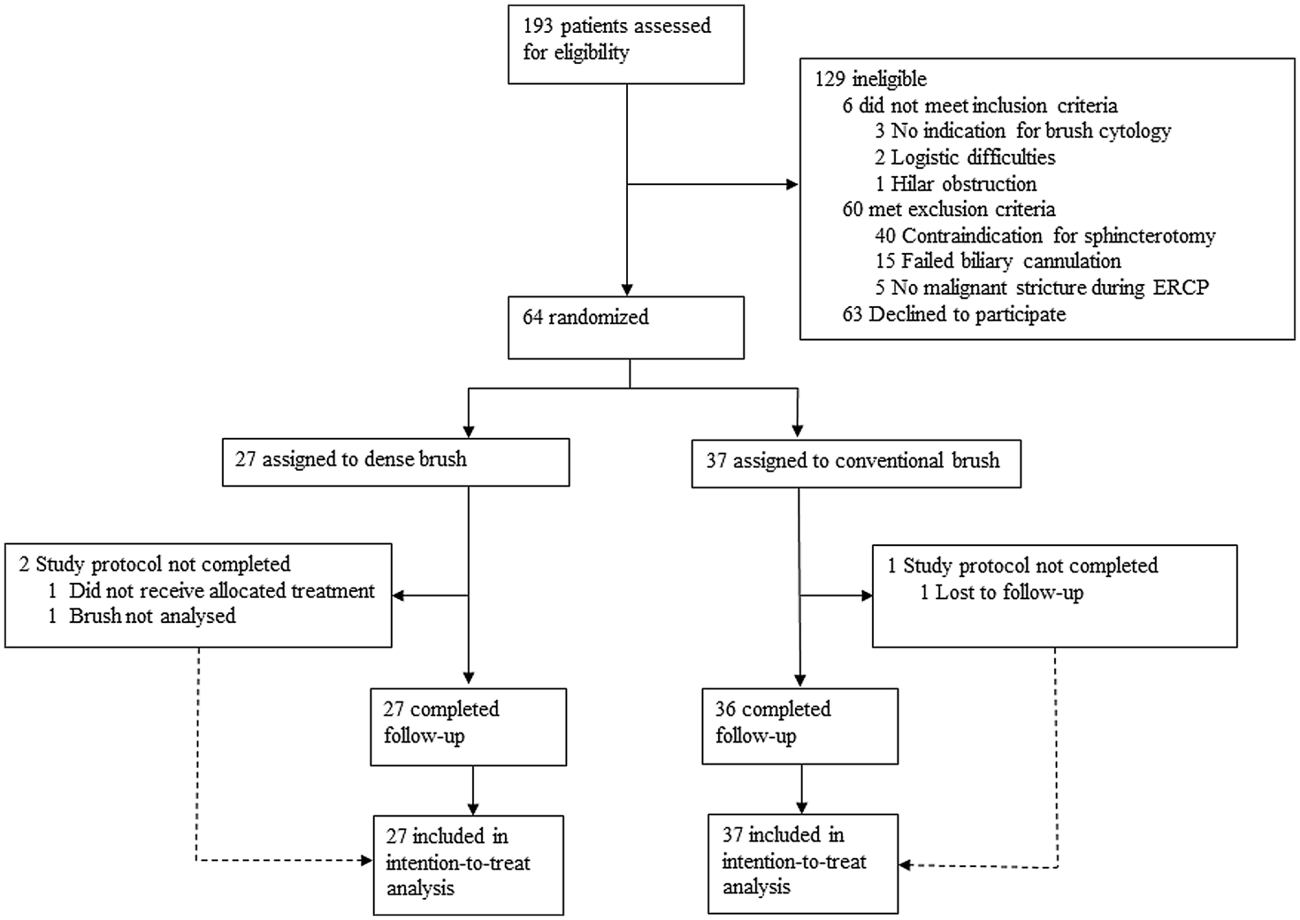
Study diagram, *ERCP* endoscopic retrograde cholangiopancreatography

We assessed 193 patients for participation between June 13th, 2016 and June 15th, 2021, of whom 64 patients were eligible for inclusion (Fig. 1). The cohort had a median age of 69 years (IQR 61–75 years) and 39 patients (61%) were male. Patients were randomized to the intervention group (*n* = 27, 42%) or the control group (*n* = 37, 58%). Because of the randomized study design, baseline and disease characteristics were considered similar among the groups (Table 1 and Table S1). Endoscopic sphincterotomy was performed significantly more often in the dense brush group (*n* = 27 [100%] versus *n* = 28 [76%], *p* = 0·008, Supplementary table S2). In total, 3 protocol violations occurred. In the dense brush group, one brush could not be analysed due to logistic issues, and one patient did not receive the allocated treatment because the dense brush could not be advanced through the stricture. In the control group, one patient was lost to follow-up after ERCP (Fig. 1). In patients with final malignant diagnosis, non-diagnostic test results occurred in 2 patients (8%) in the dense brush group and in 1 patient (3%) in the conventional brush group (Supplementary table S3). Brush cytology results showed ‘suspicious for malignancy’ or ‘malignant’ in 5 patients (19%) and 8 patients (31%) in the dense brush group, respectively, compared to 6 patients (18%) and 9 patients (27%) in the conventional brush group. Bethesda scores did not differ between the two groups (*p* = 0·84, Supplementary table S3). For a cut-off value of Bethesda ≥ 4 (‘suspicious for malignancy’), the dense brush reached a sensitivity of 50% compared to 44% for the conventional brush (*p* = 0·785, Table 2). Specificity, PPV, and NPV were 100%, 100%, and 7% for the dense brush and 100%, 100%, and 14% for the conventional brush, respectively.

**Table 1:** Baseline and disease characteristics

	Dense brush *n* = 27	Conventional brush *n* = 37
Male, *n* (%)	17 (63)	22 (60)
Age (median in years, IQR)	68 (61–74)	70 (61–77)
History of acute pancreatitis, *n* (%)	4 (15)	1 (3)
Use of drugs, *n* (%)		
Coumarins	3 (11)	2 (5)
Antiplatelet agents	5 (19)	6 (16)
NSAID	1 (4)	3 (8)
Corticosteroids	1 (4)	–
Serum bilirubin (median in µmol/L, IQR)	221 (95–437)	249 (169–341)
Serum ALP (median in U/L, IQR)	642 (310–910)	451 (340–634)
Serum GGT (median in U/L, IQR)	805 (449–1256)	609 (240–1057)
Final diagnosis, *n* (%)		
Pancreatic cancer	15 (56)	30 (81)
Cholangiocarcinoma	7 (26)	4 (11)
pNET	2 (7)	–
Gallbladder cancer	1 (4)	–
Ampullary carcinoma	1 (4)	–
Benign	1 (4)^a^	3 (8)^b^

*ALP* alkaline phosphatase, *GGT* gamma-glutamyl transferase, *IQR* interquartile range, *N* number, *NSAID* non steroid anti-inflammatory drug, *U/L* units per liter, *µmol/L* micromole per liter

Percentages might not sum to 100% because of rounding

^a^No classifying diagnosis was established in this patient

^b^Two patients had minor inflammatory changes and one patient had chronic cholecystitis

**Table 2:** Diagnostic accuracy for Bethesda ≥ 4

	Dense brush	Conventional brush	*p*-value
Patients with positive test			1·000^a^
TP	13	15	
FP	0	0	
Patients with negative test			1·000^a^
TN	1	3	
FN	13^b^	19^c^	
Sensitivity	50%	44%	0·785
Specificity	100%	100%	1·000^a^
PPV	100%	100%	1·000^a^
NPV	7%	14%	1·000^a^

*FN* false negative, *FP* false positive, *NPV* negative predictive value, *PPV* positive predictive value, *TN* true negative, *TP* true positive

^a^Fisher’s exact test was used

^b^Two patients had a non-representative, non-diagnostic test result, whereas in one patient the brush was not analyzed

^c^One patient had a non-representative, non-diagnostic test result

Overall adverse events and procedure-related events occurred in 20 patients (31%) and 14 patients (22%), respectively. Its incidence was similar among the groups (Table 3)*.* Pancreatitis occurred in 5 patients (19%) in the dense brush group, compared to 2 patients (5%) in the conventional brush group (*p* = 0·13). Three patients (5%) deceased within 30 days after the procedure because of disease progression, 2 (7%) in the dense brush group and 1 (3%) in the conventional brush group (*p* = 0·57).

**Table 3:** Serious adverse events

	Dense brush *n* = 27	Conventional brush *n* = 37	*p*-value
Pancreatitis, *n* (%)	5 (19)	2 (5)	0·13^a^
Bleeding, *n* (%)	–	1 (3)	1·00^a^
Cholangitis, *n* (%)	–	2 (5)	0·50^a^
Cholecystitis, *n* (%)	–	1 (3)	1·00^a^
Stent dysfunction, *n* (%)	1 (4)	1 (3)	1·00^a^
Post-procedural pain, *n* (%)	–	3 (8)	0·26^a^
Other^b^, *n* (%)	1 (4)	3 (8)	0·63^a^
Overall adverse events, *n* (%)	7 (26)	13 (35)	0·43
Procedure-related SAE^c^, *n* (%)	6 (22)	7 (19)	0·75
Clavien–Dindo score ≥ 3, *n* (%)	1 (4)	4 (11)	0·58^a^

*N* number, *SAE* serious adverse event

Percentages might not sum to 100% because of rounding

^a^Fisher’s exact test was used

^b^One patient suffered from dehydration, one from diarrhea after chemotherapy, one experienced delayed gastric emptying, and one patient developed duodenal obstruction

^c^Clinically relevant gastro-intestinal bleeding, pancreatitis, cholangitis, cholecystitis or stent dysfunction which required hospitalization were considered as procedure-related SAE

## Discussion

This randomized controlled trial compared the sensitivity of two intraductal brush cytology devices and showed that the dense brush was not superior to the conventional brush in diagnosing malignant extrahepatic biliary strictures. Following the recommendation from the DSMB, this study was interrupted after the interim analysis for reasons of futility.

Several studies have investigated the sensitivity of brush cytology devices in identifying malignant extrahepatic biliary strictures. The modest sensitivity (44–50%) in our study is in line with results from two systematic reviews that reported sensitivity rates of 42% (± 3.2%) and 45% (95%-CI 40–50%), respectively. [5, 6] A previously published randomized controlled trial investigating the sensitivity of a conventional versus a dense brush showed similar results when compared to our study [16]. In contrast to our study, the sensitivity of both brushes was not clearly mentioned in their manuscript. Their study design also differed from our study, since Kylänpää et al. obtained brush cytology samples by advancing the brushing device through the biliary tract 5 times only. This might have influenced the results, especially since a recent randomized controlled trial showed that more passes (30 times) reached higher sensitivity when compared to 10 or 20 passes, although the performance of prior dilatation was not reported in that study [17]. Interestingly, we observed a trend towards a higher rate of pancreatitis in the dense brush group, in contrast to Kylänpäa et al., who reported a higher rate of hyperamylasemia in the conventional brush group [16]. This finding might originate from the higher proportion of patients with a history of acute pancreatitis (15%) in the dense brush group in our cohort. Future studies focusing specifically on adverse events after biliary brush cytology are required to draw a definite conclusion on this matter.

In addition, three retrospective studies showed conflicting results on the increased sensitivity of dense brush cytology devices. Two of these studies did show higher sensitivity for the dense brush when compared to conventional brush cytology devices, although Bank et al. did not observe a higher sensitivity [7, 8, 18]. However, apart from their retrospective design, these studies compared the results of the dense brush to a historical cohort, thereby introducing the risk of historical bias. Although previous studies report conflicting results as to which malignant etiology yields highest brush sensitivity, our results might have been influenced by the heterogeneity in the distribution of etiologies (e.g., PDAC, CCA, ampullary cancers) between the two groups [19, 20]. Nevertheless, a recent meta-analysis reported a 56% sensitivity for brush cytology in 1123 CCA patients, thereby underlining that the sensitivity of brush cytology is also modest in CCA [21]. It is well known that EUS-guided tissue acquisition has a superior sensitivity to diagnose malignancy (100% as reported by a recent Cochrane review) when compared to brush cytology, especially in patients with PDAC [22]. The role of brush cytology nevertheless remains crucial as performing a single-session EUS and ERCP procedure introduces logistic difficulties since it requires an endoscopist skilled in both techniques. Other patient characteristics (i.e., age and bilirubin level) are unlikely to have caused any significant confounding effect because of the randomized design of the current study [23–25]. One factor that however should be taken into account when interpreting our results, is that inter observer variability might have influenced the outcomes since central reading was not performed. [26, 27] The current study identified that new diagnostic approaches are needed to increase sensitivity in diagnosing malignant extrahepatic biliary. A promising development is next-generation sequencing on brush cytology samples, showing sensitivity rates of 83% according to a recent prospective trial by Singhi et al. [28] In addition, although a systematic review in 2015 reported a modest sensitivity (48%, 95%CI 43%–53%) for intraductal biliary biopsies, more recent studies reported favorable results with sensitivity rates of up to 81%. [5, 29, 30]

The results of this randomized study should be interpreted in light of some limitations. First, this study was ended prematurely as recommended by the DSMB for reasons of futility. As a consequence, the sample size calculated for this study was not accomplished, thereby implicating the robustness of the conclusions that can be drawn from our data. Second, it is impossible to draw any conclusions regarding the equality of both brushes since this trial was designed as a superiority trial. In addition, because block randomization was not used in this study, the number of patients allocated to each group appears unequal (*n* = 27 vs. *n* = 37). However, it is not likely that this difference has influenced study outcomes. Third, the reported sensitivity might have been influenced by the fact that 10 brush passes were performed. It is however unlikely that this introduced bias in the comparison since the number of passes were equal in both groups. Fourth, endoscopic sphincterotomy was performed more often in the dense brush group, possibly leading to blood contamination and thus hampering cytological outcomes. It is however unlikely that this influenced the results since only 3 non-diagnostic samples were observed in the study cohort. Fifth, this study focused on patients with extrahepatic strictures and the results might therefore not be generalizable to patients with perihilar or intrahepatic strictures. Last, the specificity reported in this study is only based on the results of 4 patients and should therefore be interpreted with caution. The strengths of this study consist primarily of its prospective, randomized design. In addition, the inclusion of all consecutive patients with suspected malignant biliary strictures reflects the patient population in clinical practice. We minimalized the risk of bias potentially caused by differences in experience among dedicated interventional endoscopists by standardizing the brush cytology procedures in the study protocol. Lastly, this study also evaluated the incidence of adverse events and thereby provides a thorough overview of the benefits as well as the disadvantages of both biliary brush devices.

In conclusion, this randomized controlled trial showed that the dense brush is not superior to the conventional brush in terms of sensitivity in diagnosing malignant extrahepatic biliary strictures. As a consequence, this study was prematurely terminated for reasons of futility. Future studies should focus on the application of new techniques to evaluate biliary brush specimens (e.g., DNA mutation analysis) and on the value of advanced endoscopic procedures to obtain biliary samples (e.g., intraductal biopsies).

## Supplementary Information

Below is the link to the electronic supplementary material.

Supplementary file1 (DOCX 22 KB)

## Data Availability

The data that support the findings of this study are available from the corresponding author upon reasonable request. Individual patient data will be shared after de-identification and approval by the study team. Furthermore, a data transfer agreement has to be set up prior to data sharing.

## Abbreviations

ALP: Alkaline phosphatase
CCA: Cholangiocarcinoma
CT: Computed tomography
DNA: Deoxyribonucleic acid
DSMB: Data safety monitoring board
ERCP: Endoscopic retrograde cholangiopancreatography
EUS: Endoscopic ultrasonography
FN: False negative
FNA: Fine-needle aspiration
FP: False positive
GGT: Gamma-glutamyl transferase
IQR: Interquartile range
N: Number
N/A: Not applicable
NPV: Negative predictive value
NSAID: Non steroid anti-inflammatory drug
NTR: Netherlands trial register
PD: Pancreatic duct
PDAC: Pancreatic ductal adenocarcinoma
pNET: Pancreatic neuroendocrine tumor
PPV: Positive predictive value
SAE: Serious adverse event
SEMS: Self-expandable metal stent
TN: True negative
TP: True positive
U/L: Units per liter
µmol/L: Micromole per liter
