# Supplementary material for: Comparison of two intraductal brush cytology devices for suspected malignant biliary strictures: randomized controlled trial
Source: Surg Endosc. 2023 Feb 27;37(6):4566–73. doi: 10.1007/s00464-023-09916-9 (PMC10234858; doi:10.1007/s00464-023-09916-9)
Supplement: Supplementary file 1 — Supplementary file1 (DOCX 22 KB) [file 464_2023_9916_MOESM1_ESM.docx]

**SUPPLEMENTARIES**

| Table S1. Disease characteristics | | |
| --- | --- | --- |
|  | Dense brush  n = 27 | Conventional brush  n = 37 |
| Cause of obstruction, n (%)  Malignant  Benign | 26 (96)  1^a^ (4) | 34 (92)  3^b^ (8) |
| Determination of final diagnosis, n (%)  Clinical/radiological follow-up EUS-FNA  Percutaneous FNA metastasis  Percutaneous biopsy mass  Percutaneous biopsy metastasis  Surgical specimen | 3 (11)  5 (19)  3 (11)  1 (4)  2 (8)  13 (48) | 3 (8)  14 (38)  2 (5)  -  4 (11)  14 (38) |
| Percentages might not sum to 100% because of rounding.  ^a^ No classifying diagnosis was established in this patient. ^b^ Two patients had minor inflammatory changes and one patient had chronic cholecystitis.  Abbreviations: EUS = endoscopic ultrasonography. FNA = fine-needle aspiration. N = number. pNET = pancreatic neuroendocrine tumor. | | |

| Table S2. Procedure characteristics | | | |
| --- | --- | --- | --- |
|  | Dense brush  n = 27 | Conventional brush  n = 37 | p-value |
| Plastic endoprothesis in situ, n (%) | 4 (15) | 4 (11) | 0·71^a^ |
| Prophylactic medication, n (%)  NSAID, n (%)  Antibiotics, n (%) | 25 (93)  4 (15) | 36 (97)  3 (8) | 0·57^a^  0·44^a^ |
| PD manipulation, n (%)  Guidewire passage  Contrast injection  Both guidewire and contrast | 2 (7)  -  6 (22) | 7 (19)  1 (3)  6 (16) | 0·28^a^  1·00^a^  0·54 |
| Placement of PD stent, n (%) | 3 (11) | 4 (11) | 1·00^a^ |
| Dilation prior to brush, n (%) | 3 (11) | - | 0·07^a^ |
| Endoscopic sphincterotomy, n (%) | 27 (100) | 28 (76) | **0·008**^a^ |
| Stent type  Fully covered SEMS  Partially covered SEMS  Plastic endoprothesis | 24 (89)  1 (4)  2 (7) | 32 (87)  2 (5)  3 (8) | 1·00^a^ |
| Antibiotics after procedure, n (%) | 3 (11) | 1 (3) | 0·30^a^ |
| Per procedural complications, n (%) | 1 (4) | - | 0·42^a^ |
| Percentages might not sum to 100% because of rounding. ^a^ Fisher’s exact test was used.  Abbreviations: IQR = interquartile range. N = number. NSAID = non steroid anti-inflammatory drug. PD = pancreatic duct. SEMS = self-expandable metal stent. | | | |

| Table S3. Cytology results in patients with malignant disease | | | |
| --- | --- | --- | --- |
|  | Dense brush^a^  n = 27 | Conventional brush  n = 37 | p-value |
| DNA mutation analysis, n (%) | 1 (4) | - | 0·18^b^ |
| Immunohistochemical staining, n (%) | 2 (8) | - | 0.08^b^ |
| Cytology suspicious of malignancy, n (%)  No  Not likely  Possibly  Likely  Definitively | 4 (15)  5 (19)  3 (12)  5 (19)  8 (31) | 11 (32)  2 (6)  6 (18)  7 (21)  8 (24) | 0·35^b^ |
| Highest Bethesda score, n (%)  Not representative, non-diagnostic  Negative for malignancy  Atypical  Dysplasia, benign  Suspicious for malignancy  Malignant | 2 (8)  6 (23)  4 (15)  -  5 (19)  8 (31) | 1 (3)  12 (35)  6 (18)  -  6 (18)  9 (27) | 0·84^b^ |
| Percentages might not sum to 100% because of rounding.  ^a^ In one patient in the dense brush group the cytology results are missing due to loss of the brush cytology sample. ^b^ Fisher’s exact test was used.  Abbreviations: DNA = deoxyribonucleic acid. N = number. | | | |
